# Stage-Dependent Role of Eicosanoids in Colorectal Cancer

**DOI:** 10.3390/ijms27041641

**Published:** 2026-02-08

**Authors:** Jakub Klekowski, Paulina Fortuna, Mariusz Chabowski, Łukasz Lewandowski, Wioleta Szewczak, Karolina Mosna, Gabriela Maciejewska, Marek Zawadzki, Małgorzata Krzystek-Korpacka, Mariusz Fleszar

**Affiliations:** 1 Department of Surgery, 4th Military Clinical Hospital, 50-981 Wroclaw, Poland; mariusz.chabowski@gmail.com; 2 Department of Nursing and Obstetrics, Division of Anesthesiological and Surgical Nursing, Faculty of Health Science, Wroclaw Medical University, 50-367 Wroclaw, Poland; 3 Omics Research Center, Wroclaw Medical University, 50-368 Wroclaw, Poland; paulina.fortuna@umw.edu.pl (P.F.); wioleta.szewczak@umw.edu.pl (W.S.); karolina.mosna@umw.edu.pl (K.M.); gabriela.maciejewska@umw.edu.pl (G.M.); mariusz.fleszar@umw.edu.pl (M.F.); 4 Department of Clinical Surgical Sciences, Faculty of Medicine, Wroclaw University of Science and Technology, 50-556 Wroclaw, Poland; zawadzki@wssk.wroc.pl; 5 Department of Biochemistry and Immunochemistry, Wroclaw Medical University, 50-368 Wroclaw, Poland; lukasz.lewandowski@umw.edu.pl (Ł.L.); malgorzata.krzystek-korpacka@umw.edu.pl (M.K.-K.); 6 Research and Development Centre at Regional Specialist Hospital, 51-124 Wroclaw, Poland

**Keywords:** eicosanoids, colorectal cancer, lipidomics, prostaglandins, thromboxanes

## Abstract

Colorectal cancer (CRC) is a major health concern with increasing incidence, especially in younger adults. This study evaluated the stage-dependent role of serum eicosanoids as biomarkers in CRC patients. A cohort of 122 patients undergoing curative colorectal resection was prospectively recruited. Serum eicosanoid profiles were evaluated using targeted metabolomics and analyzed through regression-based statistical models to identify associations with CRC staging. The more advanced stages of CRC (with N+ and M+) showed significantly increased levels of PGD_2_, PGE_2_, and TXB_2_. The latter proved to be consistently associated with advanced disease. LTB_4_ and PGD_2_ showed inverse relationships relative to each other with respect to local invasion, showing PGD_2_ as a marker of higher T stages. PGE_2_ was not recognized as a viable biomarker. The progression of CRC is associated with distinct alterations in eicosanoid profiles. This study showed the potential of TXB_2_, LTB_4_, and PGD_2_ as indicators of CRC advancement.

## 1. Introduction

Colorectal cancer (CRC) is the third most common and second most lethal malignancy in the world. It is estimated to account for about 1.93 million new cases and 940,000 deaths globally in a year. There are distinct geographic and demographic differences in CRC prevalence. Although the incidence rates are the highest in high-income regions, there is a rapid increase in low- and middle-income countries, which could be linked to changing lifestyles and dietary habits. CRC remains a major public health concern in the United States. Diagnoses among adults under 50 are rising, while they are declining in older populations. This underlines the need for targeted prevention, early detection, and easy access to screening [1,2,3].

Eicosanoids are highly active lipid-derived mediators generally synthesized from 20-carbon polyunsaturated fatty acids (PUFAs). The most common substrate for eicosanoids is arachidonic acid (AA)—a cell membrane lipid—which is transformed through cyclooxygenase, lipoxygenase, and cytochrome P450 pathways. Eicosanoids exert their functions mostly at the site of their production, where they regulate numerous physiological processes, like inflammation, immunity, vascular tone, and gastrointestinal integrity. There is also evidence that they contribute to cancer pathology. Eicosanoid pathways play a crucial role in maintaining homeostasis, but in certain conditions, they drive pathological processes, therefore making them important targets for therapeutic intervention [4,5,6]. Eicosanoids also modulate the recruitment of immune cells, thus playing a vital role in immunological response to cancer and shaping the tumor microenvironment. They take part in remodeling the extracellular matrix and regulate processes such as angiogenesis and vascular permeability, which are important when considering the metastatic potential of cancer [7,8,9].

The eicosanoid signaling in the cells is generally conveyed through specific G protein-coupled receptors (GPCRs). Prostanoids, leukotrienes, hydroxyeicosanoids, and epoxyeicosatrienoic acids trigger intracellular signaling cascades. The prostanoid receptor family includes eight subtypes (EP1–EP4, DP1–DP2, FP, IP, and TP) that couple to different G proteins (Gαs, Gαq, Gαi, or Gα12/13). They regulate cAMP levels, calcium mobilization, Rho activation, and other pathways [4,10].

Due to the distinct connection of eicosanoids to immunological response and activation of inflammatory pathways, non-steroidal anti-inflammatory drugs (NSAIDs) were sought as possible anti-cancer agents. The most extensively evaluated in studies was the widely used aspirin. NSAIDs inhibit cyclooxygenase (COX) enzymes, reducing the production of prostaglandins, especially prostaglandin E_2_ (PGE_2_). Reports show that PGE_2_ promotes tumorigenesis by inhibiting apoptosis, stimulating cell proliferation and angiogenesis, and modulating immune surveillance over cancer cells. Overexpression of COX-2 was shown in the studies. Several reports present the possible use of aspirin and other NSAIDs in lowering the risk of CRC and colorectal adenomas. However, the long-term use of these drugs carries substantial risks, therefore limiting their use [11,12,13].

Prostaglandins are generally produced early in response to acute inflammation within tissues. The main function is exerted by PGE_2_, which promotes vasodilatation and permeability. If acute inflammation is not resolved in time, its state becomes chronic with constant synthesis of inflammatory cytokines—characteristic of neoplasms. PGD_2_, on the other hand, is believed to play a role in the resolution of inflammation, but at the same time, it is responsible for allergic reactions in conditions such as asthma [14,15].

Plasma and serum are accessible bodily fluids that enable the diagnosis of multiple diseases. The same applies to cancer patients and to the detection of cancer biomarkers. A recent study points out 12-lipoxygenase (12-LOX) in plasma extracellular vesicles (EVs) as a potential pro-tumorigenic factor for CRC [16]. In another article, PGE_2_ was found to be inversely associated with the risk of advanced adenoma, which stands in contrast to a general belief of its carcinogenic potential, although observed levels were lower than in CRC patients [17]. To continue, other reports showed that higher AA and PGE_2_ levels were directly linked to CRC risk [5,18].

The aforementioned articles highlight the increasing need to evaluate eicosanoids as potential biomarkers and therapeutic targets for CRC. In our study, we assessed the role of eicosanoids in the serum of CRC patients as a stage-dependent biomarker.

## 2. Results

### 2.1. Population Sample Characteristics 

The study cohort (Table 1) comprised patients stratified according to the primary outcome, i.e., TNM stage < III (n = 66) versus TNM ≥ III (n = 56). Baseline characteristics are summarized in Table 1. The two groups were largely comparable in demographic and clinical variables, with selected differences noted in age and several histopathological features. Patients with advanced disease (TNM ≥ III) tended to be younger (median: 69.0 years) compared with those at earlier stages (71.5 [66.3–77.8]; *p* = 0.046). The body mass index and tumor size did not differ between groups (median BMI ≈ 26 kg/m^2^; tumor size ≈ 40 mm, both *p* > 0.4). No significant differences were found in CEA concentrations or smoking exposure.

Among the quantified lipid mediators, PGD_2_, PGE_2_, and TXB_2_ levels were higher in the TNM ≥ III group (*p* = 0.008, 0.006, and 0.008, respectively), while other metabolites showed no significant group-wise differences.

The distribution of gender, tumor localization, histologic grade, type of resection, operative method, and comorbidities (including diabetes, hypertension, heart failure, and chronic kidney disease) was similar across groups (all *p* > 0.1).

As expected from the staging definition, higher TNM stages were accompanied by more advanced T, N, and M categories: T4 lesions were more frequent among patients with TNM ≥ III (39.3% vs. 16.7%; *p* = 0.009). Node involvement (N1/N2) occurred exclusively in the TNM ≥ III group (*p* < 0.001), as did distant metastases (M1 in 26.8% vs. 0%; *p* < 0.001).

Histopathological invasions were also enriched in advanced cases: angioinvasion (78.6% vs. 27.3%; *p* < 0.001) and neuroinvasion (39.3% vs. 15.2%; *p* = 0.005). Other molecular or immunohistochemical markers (*KRAS*, *NRAS*, *BRAF*, MSI, and MMR proteins) showed no statistically significant differences between the TNM strata.

### 2.2. Insights from Multivariate Modelling

This section is focused on statistical inference based on the models created in the process of two different strategies (penalized and likelihood-based stepwise regression). For further methodical reference, feature selection information and model diagnostics, see ‘Data Analysis Strategy’ and Supplementary Materials.

### 2.3. Primary Outcome—TNM Stage

Both models interpreted in this section are given in Table 1.

### 2.4. Ordinal Model

Under the penalized feature-selection approach (Strategy A), higher concentrations of LTB_4_ (IQR = 3.495) were modestly associated with a lower TNM stage. After adjustment for age and gender, each 3.495-unit increase in LTB_4_ corresponded to a 1.584-fold lower odds of being classified into a higher TNM category (OR = 0.631; 95% CI 0.404–0.986; *p* = 0.043). No other metabolites—including PGE_2_, PGD_2_, TXB_2_, or 15-deoxy-PGJ_2_—showed consistent or statistically significant associations in this model.

In the stepwise LRT framework (Strategy B), the strongest and most consistent signal was observed for TXB_2_. Each 8.535-unit increase in TXB_2_ was linked to a 1.312-fold higher odds of being classified into a more advanced TNM category (OR = 1.312; 95% CI 1.032–1.669; *p* = 0.027), and the effect persisted after adjustment for age and gender (OR = 1.280; 95% CI 1.001–1.639; *p* = 0.049). These findings suggest that elevated TXB_2_ is associated with overall tumor progression. 

### 2.5. Binomial Model (Odds for TNM ≥ III)

In the stepwise logistic model (Strategy B), higher TXB_2_ (IQR = 8.535) was also linked to greater odds of advanced stage: OR = 1.616 (95% CI 1.163–2.244; *p* = 0.004); after adjustment: OR = 1.560 (95% CI 1.125–2.165; *p* = 0.008). When interpreted inversely, this corresponds to a 0.619-fold (95% CI 0.446–0.860) and 0.641-fold (95% CI 0.462–0.889) lower odds of remaining in lower TNM categories per 8.535-unit rise in TXB_2_. No other metabolites showed reproducible associations in the binary models.

### 2.6. Secondary Outcomes—T and N Substages 

For the T-stage, two metabolites demonstrated opposite trends under the penalized model. A 3.495-unit increase in LTB_4_ was associated with a 2.076-fold lower odds of belonging to a higher T-category (OR = 0.481; 95% CI 0.292–0.794; *p* = 0.004), suggesting protection against local advancement. Conversely, a 0.143-unit increase in PGD_2_ was linked to a 1.876-fold higher odds of higher T-stage (OR = 1.876; 95% CI 1.096–3.205; *p* = 0.022). No consistent associations were identified in the stepwise models.

For N-stage, the stepwise model again highlighted TXB_2_ (IQR = 8.535). Each 8.535-unit increase in TXB_2_ was associated with a 1.386-fold higher odds of being in a higher N-category (OR = 1.386; 95% CI 1.083–1.773; *p* = 0.009), and the effect remained significant after adjustment (OR = 1.358; 95% CI 1.057–1.748; *p* = 0.017). Other metabolites did not show reproducible or significant patterns. Both models are shown in Table 2.

**Figure 1:**
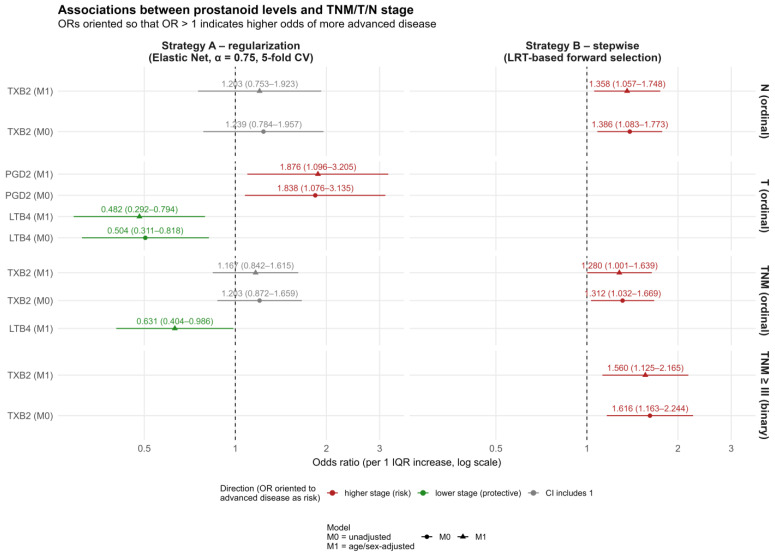
A forest-plot diagram for Strategies A and B for significant variables; TXB_2_, PGD_2_, and LTB_4_ presenting associations between their levels and CRC stage.

A graphical interpretation of these results is presented in Figure 1.

### 2.7. Exploratory Analysis Outcomes—Angio/Neuroinvasion

No metabolites were retained during feature selection for these outcomes (see Supplementary Materials).

### 2.8. Interpretation Summary

Collectively, TXB_2_ was consistently associated with both overall and nodal disease progression, suggesting its potential as a marker of advanced tumor biology. LTB_4_ and PGD_2_ were inversely related to each other, exhibiting stage-specific relationships with local invasion, which possibly reflects distinct prostanoid-mediated mechanisms at different stages of tumor spread. The coherence of these associations across unadjusted and adjusted models supports their biological plausibility despite methodological differences in feature selection.

## 3. Discussion

### 3.1. The Impact of PGE_2_ and COX-2

PGE_2_ is probably the best studied risk factor for CRC among all eicosanoids. Our study showed that in higher stages of CRC, the PGE_2_ serum level is significantly elevated. This finding corresponds with previous results and with our general understanding of this lipokine [19,20,21,22,23,24,25]. Introducing PGE_2_ in mice promotes CRC metastasis [26]. Zhang et al. studied several products of AA by evaluating its metabolome in 37 CRC patients. PGE_2_ proved to have 91% sensitivity to distinguish CRC patients from healthy controls. Additionally, PGF_2_α was also 81% specific in separating controls from the study group. PGE_2_, PGF_2_α, PGA_2_, and 15-keto-PGE_2_ were increased in CRC [27]. Although this study concurs with our results with respect to PGE_2_ elevation, PGF_2_α was not significantly different in the compared groups. However, it is important to highlight that, in this paper, a comparison was not conducted between healthy controls and disease-positive subjects. Wang et al. studied PGE_2_ levels in CRC patients and compared them to non-cancer controls. A plasma PGE_2_ level of 414.95 pg/mL showed the highest specificity at 96.36% but 19.643% sensitivity for CRC diagnosis. The generated ROC curve showed only 0.62 AUC [18]. Our study—though it showed a significant increase in PGE_2_ in higher stages of CRC—did not demonstrate a viable model utilizing PGE_2_ as a biomarker for CRC progression. The results somewhat explain the involvement of PGE_2_ in the metabolism of CRC. However, similarly to Wang’s study, we fail to demonstrate the role of a biomarker.

PGE_2_ expression was also previously studied in tissue samples. Kim et al. found no significant differences in its levels between normal mucosa and cancer tissue. However, it was noted that TNF-α could be an important inducer of prostaglandin expression [28]. Pathways of eicosanoid and prostaglandin expression were found to be altered in cancer-surrounding mucosa in right colon cancer [29]. Considering these findings might be interesting, with the study of Geng et al. showing the enhanced sensitivity of CRC cells to 5-fluorouracil when blocking the prostaglandin E synthase (PTGES)/PGE2 axis [30].

Apart from PGE_2_, its receptor EP4 gained interest as a target for intervention [31]. Recent studies show that targeting receptors prevents immunosuppression within the tumor, and this could be promising in enhancing response to total neoadjuvant therapy [20,32].

COX-2, as a key enzyme responsible for synthesizing eicosanoids and the one that is potentially easy to target with NSAIDs, has undergone extensive studies [33,34,35]. However, until now, available studies do not support the use of NSAIDs, especially aspirin, in long-term protection against spontaneous CRC. Promising results can be found in patients who are genetically predisposed to CRC [36,37]. However, it was shown that among patients with familial adenomatous polyposis syndrome, introducing aspirin does not effectively decrease TXA_2_ and PGE_2_ levels [38]. In our study, on the other hand, administration of ASA as a long-term medication was not associated with any of the variables. This issue was comprehensively addressed in a study by Obeidat et al., which proved that pre-diagnosis intake of aspirin does not improve survival in CRC patients [39].

### 3.2. The Importance of LTB_4_, TXB_2_, and PGD_2_

In the process of AA metabolism, 5-LOX activity results in the synthesis of leukotrienes. The alterations in this pathway were recognised in CRC [40,41]. Overexpression of 5-LOX in CRC was well established, as well as its tumor-promoting function. Increased expression of LTB_4_ and its receptor BLT_1_ was found in CRC cells. Inhibition of either ligands or receptors leads to apoptosis and reduced proliferation. The LTB_4_ inhibitor conjugated with gemcitabine showed potent anti-tumor activity in animal models [42,43]. Leukotriene receptor inhibitors are long-known drugs, such as montelukast and zafirlukast, and they are mostly used to treat asthma and allergy. However, experimental studies show that these drugs inhibit the growth of CRC cell lines [44]. Interestingly, Zhang et al. in their study concluded that abnormally elevated lipid metabolism predicts poor prognosis and that LTB4 can be used as an independent biomarker for chemotherapy sensitivity in patients with colorectal cancer [45]. However, our study showed an inverse relation between TLB_4_ serum levels and a tumor’s local advancement. The data about the serum levels of LTB_4_ is very scarce. The observed inverse correlation could be explained by increased systemic levels of LTB_4_ when there is a gradual progression of the tumor and systemic inflammatory signalling, but as the tumor’s growth progresses, the inflammatory response is concentrated within the tumor as immune-response cells infiltrate the cancer, thus limiting the systemic level of LTB_4_ [46,47].

Thromboxanes are products of COX enzyme activity and, apart from prostaglandins, are the only other products of COX known for their tumor-promoting function. TXA_2_ is the original product of the COX pathway, but it is rapidly hydrolyzed to TXB_2_ in a non-enzymatic way. Thromboxanes are generally derived from activated platelets and exert functions such as angiogenesis, myofibroblast proliferation, and migration. Similarly, in CRC, TXA_2_ is observed to promote migration, proliferation, and differentiation of cancer cells [40,48,49,50]. However, in an experimental in vitro study, it was found that CRC cells have strong baseline capabilities to synthesize TXB_2,_ and this ability is not induced by platelet-derived extracellular vesicles [51]. On the other hand, Gottschall et al. found an abundance of eicosanoids in CRC tissues, but in individuals treated with ASA, the levels of eicosanoids—including TXB_2_, LTB_4_, and PGD_2_ were significantly lower [52]. Our study showed good model adjustments for TXB_2_ as a marker of nodal advancement or distant spread of disease.

The role of PGD_2_ in cancer development has not been well recognized. PGD_2_ and especially its receptors were studied by Dash et al. Their research established that advanced-stage tumors express more DP2 receptors [53]. In another study, a deregulation in the levels of PGE_2_/PGD_2_ was observed. Most tumors had decreased levels of PGE_2_/PGD_2_, which prevented the switch from LTB_4_ to lipoxin and the resolution of inflammation [54]. In this study, we have shown that increased serum levels of PGD_2_ are associated with higher odds of a tumor having a higher T stage. Previously, a few studies showed significant associations between PGD_2_ and CRC or identified this prostaglandin as a potential biomarker.

### 3.3. Study Limitations

Several limitations could be recognized in this study. Most importantly, a control group was not used in this research, limiting the comparison and the broader scope of results. Secondly, some clinicopathological data (such as *KRAS*, *NRAS*, *BRAF* or MSI) were available only for a fraction of individuals; therefore, the numbers for detailed analysis could not be reached. A post-op analysis of the serum and later follow-up could provide a beneficial addition to the understanding of the presented results.

Given the limited sample size and the events-per-variable ratio, the stability of the feature selection may be limited. Although the use of two methods strengthens the results, the variability in the selected factors cannot be fully prevented.

Considering the limitations of this study, we plan to expand its scope by including tissue samples and a control group in the continuation of this research.

## 4. Materials and Methods

### 4.1. Studied Group and Material

The studied group was prospectively recruited among patients qualified for colorectal resections due to CRC in the 4th Military Clinical Hospital in Wroclaw, Poland. All patients participating in the study gave their written consent to be included. Anthropometric, demographic, and clinical data were collected based on patients’ medical history. The blood samples were collected from every patient prior to the surgical procedure. The blood samples were centrifuged at 3000 rpm for 10 min in the hospital’s laboratory, and the separated serum was carefully aspirated and immediately stored in a −80 °C freezer. After gathering the studied group, the biological material was subjected to biochemical analysis in the laboratories of the Omics Research Center of Wroclaw Medical University.

The data collected included gender; age; body weight and height; body mass index (BMI); histological type of tumor; tumor localization; neoadjuvant treatment; histological G type; tumor’s size; the TNM stage (according to the AJCC 8th Edition); tumor’s angioinvasion and neuroinvasion; *KRAS*, *NRAS* and *BRAF* mutations; microsatellite instability (MSI) evaluated by immunohistochemistry (IHC) including MLH1, MSH2, MSH6 and PMS2 or genetical tests; and preoperative carcinoembryonic antigen (CEA) levels. The past medical history of patients and their current diseases, as well as the most common drugs, were also included in order to identify potential disturbing factors. The clinicopathological data are summarized in Table 3.

For this study, 122 patients were included. The inclusion criteria were age >18 years old; qualification for curative colorectal resection due to a colorectal tumor confirmed as stage I–IV; and elective surgery. The exclusion criteria were age <18 years old, tumor type other than cancer (for example, neuroendocrine tumor, inflammatory tumor, and non-malignant adenoma), and urgent surgery.

### 4.2. Biological Material Analysis

#### 4.2.1. Materials

Standards of Thromboxane B2, Leukotriene B4, Prostaglandin D2, Prostaglandin E2, 6-keto Prostaglandin F1α, Prostaglandin F2α, 15-deoxy-Δ12,14-Prostaglandin J2, and 13,14-dihydro Prostaglandin E1 and their isotope-labeled standards were procured from Cayman Chemical Company (Ann Arbor, MI, USA). Methanol, acetonitrile (ACN), ethyl acetate, water, and formic acid (FA) were acquired from Witko (Warsaw, Poland).

#### 4.2.2. Targeted Metabolomic Analysis

Samples were subjected to a quantitative analysis. Compounds were separated using an Xevo Absolute triple quadrupole mass spectrometer from Waters, Milford, MA, USA. Separation of eicosanoids was achieved based on the previously described method [55]. Briefly, 100 µL of samples or calibration standards, placed in 2 mL Eppendorf tubes, was mixed with 20 µL of 0.2% FA and 10 µL of internal standards in methanol for 1 min at 1100 RPM and 25 °C. Afterward, 200 µL of ACN and 250 µL of ethyl acetate were added to the samples and mixed for 10 min at 1100 RPM and 25 °C. The mixtures were centrifuged at 4 °C for 7 min at 15,000 RCF. A 370 µL aliquot of the obtained supernatant was evaporated to dryness and re-dissolved in 25 µL of 20% ACN in water before analysis.

Chromatographic separation of metabolites was conducted on a BEH Shield C18 column (100 mm × 2.1 mm i.d., 1.7 µm; Waters). Data acquisition for all compounds was carried out on MassLynx Software 4.2 SCN 10.50 (Waters) in the multiple reaction monitoring mode (MRM).

### 4.3. Data Analysis Strategy 

Data analysis was performed with R 4.4.2 (packages: VGAM, ordinalNet, glmnet, MASS, brant, and openxlsx). Statistical inference was based on the frequentist approach with α = 0.05.

Population sample characteristics were based on medians with 1st and 3rd quartiles (quantitative features) or counts and % (qualitative/ordinal features). Group-wise comparisons were tested with the Mann–Whitney U test or chi-square test, depending on the feature type.

### 4.4. Multivariate Modeling 

Unlike commonly used projection methods such as PLS-DA, which can overstate group separation under strong multicollinearity, we prioritized model transparency and inferential robustness by employing regression-based frameworks with explicit variable selection and penalization. We modelled colorectal-cancer staging components:

- Ordinal outcomes: TNM, T, and N.
- Binary endpoints (TNM ≥ III, angioinvasion, and neuroinvasion).

The TNM stage was the primary outcome, while T and N were secondary outcomes; angioinvasion and neuroinvasion were exploratory analyses. To improve stability with small samples, sub-stages were collapsed a priori: TNM (e.g., IA/IB/IC → I; IIA/IIB/IIC → II; IIIA/IIIB/IIIC → III; IVA/IVB → IV); T (0 → 1; 4a/4b → 4); N (1a/1b/1c → 1; 2a/2b → 2).

Prespecified metabolites were the primary predictors. Age and gender served as covariates in adjusted models only. Analyses used complete cases per outcome/tier. Metabolites were robust-scaled (median/IQR); age and gender were left unscaled. Ordinal predictors were coded as ordered factors; nominal predictors were coded as unordered. 

Ordinal endpoints used proportional-odds cumulative logit models, while binary endpoints used logistic regression. Feature selection was performed on metabolite-only models (M0); then, the coefficients were refit without penalties on the same scale. The models with adjustments (M1) additionally included age and gender. Differences in fit between M0 and M1 were tested by likelihood ratio tests (LRT). Effects are reported as odds ratios (OR) with 95% CIs. For interpretability, odds ratios are reported using a unified orientation in the main tables, while the native parameterization of the ordinal models is retained in the Supplementary Materials.

Feature selection was based on two distinct strategies:

- Penalized regression (Strategy A):
  (a) Elastic net regularized proportional odds model (cumulative logit link, α = 0.75, 5-fold CV, 1-SE rule; ranking by sum of |β| across thresholds);
  (b) Elastic net binomial model (logit link, α = 0.75, 5-fold stratified CV, λ.1se; ranking by |β|).

For both (a) and (b), the number of selected metabolites was capped at 4.

- Likelihood-based stepwise regression (Strategy B): Bidirectional LRT with enter *p* < 0.05/stay *p* < 0.10 (max 100 steps) on scaled data. Final sets were refit without penalty.

These two complementary strategies were used to evaluate the robustness of metabolite selection under different regularization paradigms. Both strategies were treated as sensitivity analyses of variable selection.

For all ordinal models (M0 and M1), the proportional odds assumption was evaluated using the Brant test, yielding no issues needing attention. 

In the Supplementary Materials, for each outcome, we provide the following: selected metabolites (ranked; raw vs. cap for A), M0/M1 coefficient tables, LRT for M1 vs. M0, Brant results (ordinal), and the scaling log (median/IQR). For the stepwise regression feature selection strategy, we additionally report the stepwise history. Given the limited events-per-variable ratio, bootstrap resampling was not applied, as it would yield unstable estimates and frequent convergence issues.

## 5. Conclusions

Our study indicates that CRC progression is associated with distinct changes in eicosanoid profiles, with TXB_2_ emerging as consistently correlated with advanced disease stages and nodal involvement, along with PGD_2_ implicating higher local advancement, while LTB_4_ shows stage-specific inverse relationships with local invasion. These findings highlight the potential of eicosanoids as indicators for colorectal cancer staging.

## Figures and Tables

**Table 1:** Ordinal and binary logistic regression models for TNM stage under two feature-selection strategies: Strategy A—penalized elastic net (α = 0.75, 5-fold CV); Strategy B—likelihood-based stepwise LRT. Models were estimated unadjusted (M0) and adjusted (M1) for age and sex. ORs per 1 IQR increase (robust-scaled). “-” denotes non-selected variables.

Modelled: TNM (ordinal)										
Metabolite	Values used for centering		Strategy A: unadjusted (M0)		Strategy A: adjusted (M1)		Strategy B: unadjusted (M0)		Strategy B: adjusted (M1)	
	Median	IQR	OR (95% CI)	*p*	OR (95% CI)	*p*	OR (95% CI)	*p*	OR (95% CI)	*p*
X13_14_DH_PGE1	0.010	0.022	-	-	-	-	-	-	-	-
X15_DEOXY_PGJ2	0.117	0.257	1.171 (0.911–1.504)	0.219	1.250 (0.929–1.683)	0.141	-	-	-	-
X6_KETO_PGF1ALPHA	0.028	0.029	-	-	-	-	-	-	-	-
LTB4	0.688	3.495	0.672 (0.437–1.036)	0.072	**0.631 (0.404–0.986)**	**0.043**	-	-	-	-
PGD2	0.084	0.143	-	-	-	-	-	-	-	-
PGE2	0.074	0.126	1.130 (0.869–1.468)	0.362	1.122 (0.862–1.462)	0.392	-	-	-	-
PGF2ALPHA	0.104	0.151	-	-	-	-	-	-	-	-
TXB2	4.528	8.535	1.203 (0.872–1.659)	0.259	1.167 (0.842–1.615)	0.355	**1.312 (1.032–1.669)**	**0.027**	**1.280 (1.001–1.639)**	**0.049**
Modelled: TNM ≥ III (binomial)										
Metabolite	Values used for centering		Strategy A: unadjusted (M0)		Strategy A: adjusted (M1)		Strategy B: unadjusted (M0)		Strategy B: adjusted (M1)	
	Median	IQR	OR (95% CI)	*p*	OR (95% CI)	*p*	OR (95% CI)	*p*	OR (95% CI)	*p*
X13_14_DH_PGE1	0.010	0.022	-	-	-	-	-	-	-	-
X15_DEOXY_PGJ2	0.117	0.257	-	-	-	-	-	-	-	-
X6_KETO_PGF1ALPHA	0.028	0.029	-	-	-	-	-	-	-	-
LTB4	0.688	3.495	-	-	-	-	-	-	-	-
PGD2	0.084	0.143	-	-	-	-	-	-	-	-
PGE2	0.074	0.126	-	-	-	-	-	-	-	-
PGF2ALPHA	0.104	0.151	-	-	-	-	-	-	-	-
TXB2	4.528	8.535	-	-	-	-	**1.616 (1.163–2.244)**	**0.004**	**1.560 (1.125–2.165)**	**0.008**

Odds ratios (ORs) are reported per 1 IQR increase in metabolite concentration after robust scaling (median/IQR) and oriented so that OR > 1 indicates higher odds of more advanced disease (higher TNM category or TNM ≥ III). In the Supplementary Materials, results from POLR are presented using the native parameterization of that tool, which implies the opposite OR orientation.

**Table 2:** Ordinal logistic regression models for T and N stages under two feature-selection strategies: Strategy A—penalized elastic net (α = 0.75, 5-fold CV); Strategy B—likelihood-based stepwise LRT. Models were estimated unadjusted (M0) and adjusted (M1) for age and sex. ORs per 1 IQR increase (robust-scaled). “-” denotes non-selected variables.

Modelled: T (ordinal)										
Metabolite	Values used for centering		Strategy A: unadjusted (M0)		Strategy A: adjusted (M1)		Strategy B: unadjusted (M0)		Strategy B: adjusted (M1)	
	Median	IQR	OR (95% CI)	*p*	OR (95% CI)	*p*	OR (95% CI)	*p*	OR (95% CI)	*p*
X13_14_DH_PGE1	0.010	0.022	-	-	-	-	-	-	-	-
X15_DEOXY_PGJ2	0.117	0.257	1.297 (0.966–1.742)	0.083	1.349 (0.971–1.880)	0.075	-	-	-	-
X6_KETO_PGF1ALPHA	0.028	0.029	-	-	-	-	-	-	-	-
LTB4	0.688	3.495	**0.504 (0.311–0.818)**	**0.006**	**0.482 (0.292–0.794)**	**0.004**	-	-	-	-
PGD2	0.084	0.143	**1.838 (1.076–3.135)**	**0.026**	**1.876 (1.096–3.205)**	**0.022**	-	-	-	-
PGE2	0.074	0.126	-	-	-	-	-	-	-	-
PGF2ALPHA	0.104	0.151	-	-	-	-	-	-	-	-
TXB2	4.528	8.535	0.728 (0.469–1.127)	0.155	0.706 (0.453–1.101)	0.125	-	-	-	-
Modelled: N (ordinal)										
Metabolite	Values used for centering		Strategy A: unadjusted (M0)		Strategy A: adjusted (M1)		Strategy B: unadjusted (M0)		Strategy B: adjusted (M1)	
	Median	IQR	OR (95% CI)	*p*	OR (95% CI)	*p*	OR (95% CI)	*p*	OR (95% CI)	*p*
X13_14_DH_PGE1	0.010	0.022	-	-	-	-	-	-	-	-
X15_DEOXY_PGJ2	0.117	0.257	-	-	-	-	-	-	-	-
X6_KETO_PGF1ALPHA	0.028	0.029	-	-	-	-	-	-	-	-
LTB4	0.688	3.495	0.811 (0.522–1.259)	0.351	0.807 (0.521–1.253)	0.341	-	-	-	-
PGD2	0.084	0.143	-	-	-	-	-	-	-	-
PGE2	0.074	0.126	1.095 (0.836–1.435)	0.511	1.091 (0.832–1.431)	0.530	-	-	-	-
PGF2ALPHA	0.104	0.151	1.102 (0.744–1.634)	0.627	1.116 (0.747–1.667)	0.591	-	-	-	-
TXB2	4.528	8.535	1.239 (0.784–1.957)	0.358	1.203 (0.753–1.923)	0.437	**1.386 (1.083–1.773)**	**0.009**	**1.358 (1.057–1.748)**	**0.017**

Odds ratios (OR) are reported per 1 IQR increase in metabolite concentration after robust scaling (median/IQR) and oriented so that OR > 1 indicates higher odds of more advanced disease (higher T or N category). In the Supplementary Materials, results from POLR are presented using the native parameterization of that tool, which implies the opposite OR orientation.

**Table 3:** Population sample characteristics.

Quantitative features					
Variable	TNM < III		TNM ≥ III		*p* (Mann–Whitney U)
	Median [Q1–Q3]	Mean (SD)	Median [Q1–Q3]	Mean (SD)	
Age	71.500 [66.250–77.750]	70.833 (10.200)	69.000 [58.500–74.000]	66.893 (11.223)	**0.046**
BMI	26.680 [23.678–28.080]	26.610 (4.456)	26.330 [22.318–29.460]	26.382 (4.998)	0.823
Tumor size	40.000 [30.000–45.000]	39.030 (14.815)	40.000 [30.000–50.000]	41.036 (15.347)	0.456
Cigarettes [package years] if smoking	30.000 [17.000–40.000]	30.280 (13.719)	25.000 [10.000–40.000]	29.227 (20.683)	0.485
CEA	2.490 [1.688–5.465]	6.843 (13.056)	3.520 [1.675–16.220]	20.574 (45.359)	0.200
X13_14_DH_PGE1	0.008 [0.000–0.021]	0.014 (0.018)	0.011 [0.002–0.023]	0.015 (0.015)	0.535
X15_DEOXY_PGJ2	0.102 [0.044–0.302]	0.192 (0.236)	0.131 [0.048–0.310]	0.322 (0.659)	0.318
X6_KETO_PGF1ALPHA	0.025 [0.016–0.040]	0.038 (0.048)	0.031 [0.020–0.051]	0.044 (0.042)	0.107
LTB4	0.540 [0.266–2.941]	2.334 (3.488)	0.942 [0.393–3.871]	2.605 (3.173)	0.150
PGD2	0.065 [0.025–0.119]	0.111 (0.158)	0.099 [0.048–0.244]	0.184 (0.189)	**0.008**
PGE2	0.056 [0.030–0.123]	0.110 (0.165)	0.098 [0.041–0.285]	0.207 (0.257)	**0.006**
PGF2ALPHA	0.084 [0.043–0.167]	0.136 (0.150)	0.129 [0.053–0.250]	0.255 (0.319)	0.053
TXB2	3.626 [0.919–6.746]	5.698 (7.432)	6.602 [1.780–17.306]	12.727 (15.145)	**0.008**
Qualitative features					
Variable	Level	TNM < III n (%)	TNM ≥ III n (%)	*p* (Chi-square)	
Gender	female	36 (54.5%)	25 (44.6%)	0.364	
	male	30 (45.5%)	31 (55.4%)		
Tumor type	adenocarcinoma	54 (81.8%)	46 (82.1%)	0.533	
	mucinous adenocarcinoma or adenocarcinoma with mucinous component	12 (18.2%)	9 (16.1%)		
	complete pathological response	0 (0%)	1 (1.8%)		
Tumor localization	cecum	9 (13.6%)	10 (17.9%)	0.887	
	ascending colon	12 (18.2%)	10 (17.9%)		
	hepatic flexure	3 (4.5%)	3 (5.4%)		
	transverse colon	2 (3%)	0 (0%)		
	splenic flexure	1 (1.5%)	0 (0%)		
	descending colon	1 (1.5%)	1 (1.8%)		
	sigmoid colon	23 (34.8%)	20 (35.7%)		
	rectum	15 (22.7%)	12 (21.4%)		
Preoperative radiotherapy	0	58 (87.9%)	51 (91.1%)	0.783	
	1	8 (12.1%)	5 (8.9%)		
Preoperative chemotherapy	0	63 (95.5%)	52 (92.9%)	0.823	
	1	3 (4.5%)	4 (7.1%)		
Grade (G)	1	33 (50%)	23 (41.1%)	0.425	
	2	29 (43.9%)	31 (55.4%)		
	3	4 (6.1%)	2 (3.6%)		
Extent of resection	right hemicolectomy	26 (39.4%)	21 (37.5%)	0.844	
	left hemicolectomy	2 (3%)	1 (1.8%)		
	anterior rectal resection	25 (37.9%)	20 (35.7%)		
	abdominoperineal resection	2 (3%)	3 (5.4%)		
	sigmoidectomy	10 (15.2%)	8 (14.3%)		
	pancolectomy	1 (1.5%)	3 (5.4%)		
R-status	R0	63 (96.9%)	49 (87.5%)	0.096	
	R1	1 (1.5%)	6 (10.7%)		
	R2	1 (1.5%)	1 (1.8%)		
Operation method	laparoscopic	16 (24.2%)	10 (17.9%)	0.525	
	open	50 (75.8%)	46 (82.1%)		
Acetylsalicylic acid	no	58 (87.9%)	50 (89.3%)	>0.999	
	yes	8 (12.1%)	6 (10.7%)		
Diabetes mellitus (DM)	no	48 (72.7%)	45 (80.4%)	0.439	
	t2dm	18 (27.3%)	11 (19.6%)		
hypertension	no	20 (30.3%)	24 (42.9%)	0.211	
	yes	46 (69.7%)	32 (57.1%)		
Heart failure	no	56 (84.8%)	53 (94.6%)	0.146	
	yes	10 (15.2%)	3 (5.4%)		
Ischemic heart disease	no	51 (77.3%)	48 (85.7%)	0.339	
	yes	15 (22.7%)	8 (14.3%)		
Arrhythmia	no	53 (80.3%)	45 (80.4%)	>0.999	
	yes	13 (19.7%)	11 (19.6%)		
Hyperlipidemia	no	47 (71.2%)	39 (69.6%)	>0.999	
	yes	19 (28.8%)	17 (30.4%)		
Asthma	no	63 (95.5%)	54 (96.4%)	>0.999	
	yes	3 (4.5%)	2 (3.6%)		
copd	no	63 (95.5%)	53 (94.6%)	>0.999	
	yes	3 (4.5%)	3 (5.4%)		
Chronic kidney disease	no	59 (89.4%)	55 (98.2%)	0.111	
	yes	7 (10.6%)	1 (1.8%)		
Thyroid illness	none	58 (87.9%)	53 (94.6%)	0.333	
	hypothyroidism	7 (10.6%)	2 (3.6%)		
	hyperthyroidism	1 (1.5%)	1 (1.8%)		
Liver illness	no	65 (98.5%)	56 (100%)	>0.999	
	yes	1 (1.5%)	0 (0%)		
Prostate disease	none	16 (53.3%)	21 (67.7%)	0.337	
	benign prostate hyperplasia	12 (40%)	7 (22.6%)		
	prostate cancer	2 (6.7%)	3 (9.7%)		
Smoking	none	39 (59.1%)	33 (58.9%)	0.803	
	current smoker	2 (3%)	3 (5.4%)		
	ex-smoker	25 (37.9%)	20 (35.7%)		
Metformin	no	45 (68.2%)	44 (78.6%)	0.279	
	yes	21 (31.8%)	12 (21.4%)		
ACEI	no	42 (63.6%)	32 (57.1%)	0.585	
	yes	24 (36.4%)	24 (42.9%)		
Sartans	no	56 (84.8%)	52 (92.9%)	0.272	
	yes	10 (15.2%)	4 (7.1%)		
Statins	no	34 (51.5%)	35 (62.5%)	0.300	
	yes	32 (48.5%)	21 (37.5%)		
Ezetimibe	no	63 (95.5%)	53 (94.6%)	>0.999	
	yes	3 (4.5%)	3 (5.4%)		
B-blockers	no	37 (56.1%)	32 (57.1%)	>0.999	
	yes	29 (43.9%)	24 (42.9%)		
A-blockers	no	57 (86.4%)	50 (89.3%)	0.831	
	yes	9 (13.6%)	6 (10.7%)		
Angioinvasion	no	48 (72.7%)	12 (21.4%)	**<0.001**	
	yes	18 (27.3%)	44 (78.6%)		
Neuroinvasion	no	56 (84.8%)	34 (60.7%)	**0.005**	
	yes	10 (15.2%)	22 (39.3%)		
*KRAS* mutation	negative	4 (66.7%)	12 (60%)	>0.999	
	positive	2 (33.3%)	8 (40%)		
*NRAS* mutation	negative	5 (83.3%)	19 (95%)	0.946	
	positive	1 (16.7%)	1 (5%)		
*BRAF* mutation	negative	6 (100%)	16 (80%)	0.585	
	positive	0 (0%)	4 (20%)		
MSI	Genetic test: negative	2 (3.8%)	6 (12.5%)	0.209	
	Genetic test: positive	0 (0%)	1 (2.1%)		
	IHC: low probability	39 (75%)	35 (72.9%)		
	IHC: high probability	11 (21.2%)	6 (12.5%)		
MLH1 expression (IHC)	negative	10 (20%)	4 (10%)	0.313	
	positive	40 (80%)	36 (90%)		
MSH2 expression (IHC)	negative	1 (2%)	0 (0%)	>0.999	
	positive	49 (98%)	40 (100%)		
MSH6 expression (IHC)	negative	1 (2%)	0 (0%)	>0.999	
	positive	49 (98%)	41 (100%)		
PMS2 expression (IHC)	negative	10 (20%)	6 (14.6%)	0.695	
	positive	40 (80%)	35 (85.4%)		
TNM stage	I	16 (24.2%)	0 (0%)	-	
	II	50 (75.8%)	0 (0%)		
	III	0 (0%)	41 (73.2%)		
	IV	0 (0%)	15 (26.8%)		
T stage	T0/T1	9 (13.6%)	1 (1.8%)	**0.009**	
	T2	7 (10.6%)	5 (8.9%)		
	T3	39 (59.1%)	28 (50%)		
	T4	11 (16.7%)	22 (39.3%)		
N stage	N0	66 (100%)	2 (3.6%)	**<0.001**	
	N1	0 (0%)	33 (58.9%)		
	N2	0 (0%)	21 (37.5%)		
M stage	M0	66 (100%)	41 (73.2%)	**<0.001**	
	M1	0 (0%)	15 (26.8%)		

## Data Availability

The original contributions presented in this study are included in this article/Supplementary Materials. Further inquiries can be directed to the corresponding author(s).

## Supplementary Materials

The following supporting information can be downloaded at https://www.mdpi.com/article/10.3390/ijms27041641/s1.

## Abbreviations

The following abbreviations are used in this manuscript:

CRC	Colorectal Cancer;
AA	Arachidonic Acid;
PUFAs	Polyunsaturated Fatty Acids;
COX	Cyclooxygenase;
LOX	Lipoxygenase;
GPCRs	G Protein-Coupled Receptors;
NSAID	Non-Steroidal Anti-Inflammatory Drug;
EV	Extracellular Vesicle;
BMI	Body Mass Index;
TNM	Tumor, Node, Metastasis (Staging System);
CEA	Carcinoembryonic Antigen;
IHC	Immunohistochemistry;
MSI	Microsatellite Instability;
ASA	Acetylsalicylic Acid (Aspirin);
DM	Diabetes Mellitus;
T2DM	Type 2 Diabetes Mellitus;
ACEI	Angiotensin-Converting Enzyme Inhibitor;
PGD_2_	Prostaglandin D2;
PGE_2_	Prostaglandin E2;
TXB_2_	Thromboxane B2;
LTB_4_	Leukotriene B4;
PGF2α	Prostaglandin F2 alpha;
6-Keto-PGF1α	6-Keto Prostaglandin F1 alpha;
15-deoxy-PGJ2	15-deoxy-Δ12,14-Prostaglandin J2;
13,14-DH-PGE1	13,14-dihydro Prostaglandin E1.
