# Supplementary material for: Stage-Dependent Role of Eicosanoids in Colorectal Cancer
Source: Int J Mol Sci. 2026 Feb 8;27(4):1641. doi: 10.3390/ijms27041641 (PMC12941361; doi:10.3390/ijms27041641)
Supplement: Supplementary file 1 [file ijms-27-01641-s001.zip › Supplementary_materials.pdf]

README – Supplementary Materials

This repository contains two Excel workbooks produced by two independent modelling pipelines:

1. pipeline_multi_target.xlsx – penalised pre-selection (elastic-net/ordinalNet & glmnet) with a cap on M0 of up to 4 metabolites (see note below), followed by unpenalised refits (VGAM/GLM).
2. pipeline_stepwise_multi_target.xlsx – bidirectional stepwise selection based on LRT p-values (enter=0.05, stay=0.10), followed by unpenalised refits (VGAM/GLM).

Both pipelines analyse the same **endpoints**:

- Ordinal: TNM (merged), T (collapsed 1–4), N (collapsed 0–2)
- Binary: TNM (≥3), ANGIO (angioinvasion), NEURO (neuroinvasion)

Across both pipelines:

- M0 uses metabolites only (as selected by the respective selector).
- M1 extends M0 by age and sex (variable: cat_sexmale).
- Metabolites are robust-scaled: $(\mathrm{x} - \text{median})/\text{IQR}$computed on the analysis cohort.

The operational cap for number of selected features for M0 is 4.

File 1 — pipeline_multi_target.xlsx (penalised + cap)

Sheet inventory & how to read

- SCALING_metabolites
  Median, IQR, and N used for robust scaling of each metabolite.
  Implication: An odds ratio (OR) of a metabolite corresponds to a 1-IQR increase in the original scale.
- Brant tests (proportional odds), present when applicable
  - TNMord_Brant_M0, TNMord_Brant_M1
  - Tord_Brant_M0, Tord_Brant_M1
  - Nord_Brant_M0, Nord_Brant_M1
    Each sheet contains Brant test outputs from polr/brant. If no covariates survived selection, the sheet notes Intercept-only.
- TNM (ordinal) – selection and models
  - TNMord_selected — ranked metabolites (descending by summed $|\beta|$across thresholds) with a flag selected_cap indicating those that survived the cap (M0).
  - TNMord_selected_table — full table per metabolite: selected_raw (non-zero at 1-SE), selected_cap, score, and rank.
  - TNMord_M0_coef, TNMord_M1_coef — coefficient tables from VGAM (parallel cumulative logit): Estimate (beta-coefficient), StdErr (SE of the beta-coefficient), z, p, and exponentiated beta-coefficient with its 95% CI: OR, LCL (lower confidence interval limit), UCL (upper confidence interval limit).
  - TNMord_LRT — likelihood-ratio test comparing M1 vs M0 in terms of goodness of fit.
- TNM (binary, ≥3) – selection and models
  - TNMbin_selected, TNMbin_selected_table — analogous to ordinal, with glmnet scores at lambda.1se.

- TNMbin_M0_coef, TNMbin_M1_coef — logistic GLM coefficients with ORs.
- TNMbin_LRT_M1vM0 — LRT M1 vs M0.

- T (ordinal), N (ordinal)
  - Tord_selected/Nord_selected — ranked lists with selected_cap.
  - Tord_selected_table/Nord_selected_table — detailed selection tables.
  - Tord_M0_coef, Tord_M1_coef; Nord_M0_coef, Nord_M1_coef — VGAM coefficients and ORs.
  - Tord_LRT, Nord_LRT — LRT M1 vs M0.
- ANGIO (bin), NEURO (bin)
  - ANGIO_selected, NEURO_selected — ranked lists with selected_cap.
  - ANGIO_selected_table, NEURO_selected_table — detailed selection tables.
  - ANGIO_M0_coef, ANGIO_M1_coef; NEURO_M0_coef, NEURO_M1_coef — GLM coefficients and ORs.
  - ANGIO_LRT, NEURO_LRT — LRT M1 vs M0.

Selector details (this file):

- Ordinal: ordinalNetTune (cumulative logit), alpha=0.75, 1-SE; ranking by sum of $|\beta|$across thresholds.
- Binary: cv.glmnet (binomial), alpha=0.75, lambda.1se, stratified folds; ranking by $|\beta|$.
- Refit: unpenalised VGAM/GLM on the same scaled data.

File 2 — pipeline_stepwise_multi_target.xlsx (bidirectional stepwise LRT)

Sheet inventory & how to read

- SCALING_metabolites
  Median, IQR, N (global spec used for scaling in this pipeline).
- Brant tests (proportional odds), present when applicable
  - TNMord_Brant_M0, TNMord_Brant_M1
  - Tord_Brant_M0, Tord_Brant_M1
  - Nord_Brant_M0, Nord_Brant_M1
- TNM (ordinal) – selection and models
  - TNMord_selected — final set selected by stepwise for M0 (list only).
  - TNMord_M0_coef, TNMord_M1_coef — VGAM coefficients with ORs.
  - TNMord_LRT — LRT M1 vs M0.
  - TNMord_step_hist — full stepwise history: step, action (ADD/DROP), var, p.
- TNM (binary ≥3) – selection and models
  - TNMbin_selected — final set for M0.
  - TNMbin_M0_coef, TNMbin_M1_coef — GLM coefficients with ORs.
  - TNMbin_LRT_M1vM0 — LRT M1 vs M0.
  - TNMbin_step_hist — stepwise history.
- T (ordinal), N (ordinal)
  - Tord_selected / Nord_selected — final sets.
  - Tord_M0_coef, Tord_M1_coef; Nord_M0_coef, Nord_M1_coef — VGAM coefficients and ORs.
  - Tord_LRT, Nord_LRT — LRT M1 vs M0.
  - Tord_step_hist, Nord_step_hist — stepwise histories.
- ANGIO (bin), NEURO (bin)
  - ANGIO_selected, NEURO_selected — final sets.
  - ANGIO_M0_coef, ANGIO_M1_coef; NEURO_M0_coef, NEURO_M1_coef — GLM coefficients and ORs.
  - ANGIO_LRT, NEURO_LRT — LRT M1 vs M0.
  - ANGIO_step_hist, NEURO_step_hist — stepwise histories.

Selector details (this file):
Bidirectional stepwise with likelihood-ratio tests: enter $p<0.05$, stay $p \geq 0.10$ triggers drop; maximum 100 steps.
Refits are unpenalised (VGAM/GLM) on the same scaled data.

Conventions, variable definitions, and interpretation

- Scaling: All metabolite coefficients and ORs correspond to a 1-IQR increase (robust scaling). Age and sex are not scaled here unless specified in sheets (they are included as-is in M1).
- Endpoints:

    - ord_tnmmerged — ordinal TNM (merged).
    - ord_t_collapsed — T levels collapsed to {1,2,3,4} (4a/4b->4; 0->1).
    - ord_n_collapsed — N levels collapsed to {0,1,2} (1a/1b/1c->1; 2a/2b->2).
    - TNM (binary) — derived as TNM ≥ 3.
    - cat_angioinvasion, cat_neuroinvasion — binary; internally coerced to {0,1} if needed.
- Models:
    - Ordinal M0/M1: VGAM cumulative logit with parallel=TRUE (proportional odds). Brant sheets assess this assumption.
    - Binary M0/M1: logistic GLM.
    - LRT sheets: compare M1 vs M0 on the same cohort (after listwise deletion).
- Selection edge cases:
    - If the selector returns no metabolites, M0 becomes Intercept-only; corresponding sheets are still exported (with a note where applicable).
    - In the penalised pipeline, *_selected includes ranked lists with selected_cap (within the cap); *_selected_table exposes raw vs capped selection and scores.
    - In the stepwise pipeline, *_step_hist documents each ADD/DROP decision with LRT p-values.
- Multiple testing / inference:
  Coefficients' p-values are model-based and not adjusted for multiplicity; the exports are primarily for transparency and reproducibility, not for definitive claims per metabolite.
- Software (as used in code):
  VGAM, MASS, brant, ordinalNet, glmnet, openxlsx (R; CRAN).

Minimal reading guide for reviewers

1. Start from SCALING_metabolites to understand the scale (ORs per 1-IQR increase).
2. For each endpoint, check *_selected (and *_selected_table in the penalised file) to see which metabolites formed M0.
3. Compare *_M0_coef vs *_M1_coef to assess the impact of age/sex.
4. Use *_LRT to see whether adding age/sex (M1) improved fit over M0.
5. For ordinal endpoints, verify Brant sheets for proportional-odds diagnostics.
6. In the stepwise file, consult *_step_hist to audit the model path.

File integrity & cohorts

All analyses use listwise deletion per endpoint. As a result, cohorts may differ across endpoints and between M0 and M1 only by covariate availability; scaling specs are derived from the analysis data as noted above. Where an endpoint is missing (no rows after dropNA), the corresponding sheets are omitted.
